# A Smart Wearable Sensor System for Counter-Fighting Overweight in Teenagers

**DOI:** 10.3390/s16081220

**Published:** 2016-08-10

**Authors:** Carlo Emilio Standoli, Maria Renata Guarneri, Paolo Perego, Marco Mazzola, Alessandra Mazzola, Giuseppe Andreoni

**Affiliations:** 1 Politecnico di Milano, Dipartimento di Design, via Giovanni Durando, 38/A, 20158 Milano, Italy; mariarenata.guarneri@polimi.it (M.R.G.); paolo.perego@polimi.it (P.P.); alessandra.mazzola@polimi.it (A.M.); giuseppe.andreoni@polimi.it (G.A.); 2 Neosperience S.p.a, Corso Indipendenza 5, 20125 Milano, Italy; marco.mazzola@neosperience.com

**Keywords:** wearable sensors, smart garment design, lifestyle monitoring, teenagers, co-design

## Abstract

PEGASO is a FP7-funded project whose goal is to develop an ICT and mobile-based platform together with an appropriate strategy to tackle the diffusion of obesity and other lifestyle-related illnesses among teenagers. Indeed, the design of an engaging strategy, leveraging a complementary set of technologies, is the approach proposed by the project to promote the adoption of healthy habits such as active lifestyle and balanced nutrition and to effectively counter-fight the emergence of overweight and obesity in the younger population. A technological key element of such a strategy sees the adoption of wearable sensors to monitor teenagers’ activities, which is at the basis of developing awareness about the current lifestyle. This paper describes the experience carried out in the framework of the PEGASO project in developing and evaluating wearable monitoring systems addressed to adolescents. The paper describes the methodological approach based on the co-designing of such a wearable system and the main results that, in the first phase, involved a total of 407 adolescents across Europe in a series of focus groups conducted in three countries for the requirements definition phase. Moreover, it describes an evaluation process of signal reliability during the usage of the wearable system. The main results described here are: (a) a prototype of the standardized experimental protocol that has been developed and applied to test signal reliability in smart garments; (b) the requirements definition methodology through a co-design activity and approach to address user requirements and preferences and not only technological specifications. Such co-design approach is able to support a higher system acceptance and usability together with a sustained adoption of the solution with respect to the traditional technology push system development strategy.

## 1. Introduction

The rapidly increasing prevalence of overweight and obesity among children and adolescents reflects a global “epidemic” worldwide. Due to the associated serious medical conditions, it is estimated that obesity already accounts for up to 7% of health care costs in the EU, as well as costs to the wider economy associated with lower productivity, lost output and premature death. Obesity in younger age has been recognized as an alarming key predictor for obesity in adulthood, but also entails a number of short-term health complications at the juvenile age [1].

Obesity and overweight also have economic, social and environmental dimensions, implying direct and indirect costs. The former are connected with personal and hospital health care, health services and drugs, whereas the latter is mainly related to the reduced productivity of the obese workers. At the social level, it has been studied that the obese person often has very low self-esteem, is depressed and tends to stay on his/her own: he/she is most of the time mocked by his/her peers. Therefore, overweight people may have serious relational problems. The environment has a significant impact on the development of obesity. Many factors contribute to the sedentary lifestyle of young people and adults. As an example, the urbanization trend, the rise in the number of vehicles and the lack of cycle paths discourage parents from letting their children walk or cycle to school.

This situation is no longer sustainable: it is urgent to begin with prevention programs. Placing preventive care at the heart of health systems is of paramount importance. Advances in the understanding of health risk factors and the design of effective interventions to prevent illness are the way forward. Measuring and evaluating the quality of prevention strategies is important in order to gain a better understanding of their mechanisms of action and potential benefits and risks; to measure their impact and appropriateness; and to monitor their relevance in terms of tackling health inequalities [2].

In consideration of the above, PEGASO [3]—an FP7 project funded by the European Union (EU)—tackles prevention, focusing on obesity and related co-morbidities, in which lifestyle coaching plays a very important role. This objective is achieved by developing a platform that—leveraging mobile and ICT technologies and through wearable sensors and mobile apps—supports young people in becoming aware of risks and motivates them in a behaviour change path towards healthy lifestyles. PEGASO gives guidance towards developing good habits and provides a social platform to stimulate young people’s willingness to engage actively in their health management.

The challenge of PEGASO is to develop a system that meets the requirements of the users by adopting a user-centred design (UCD) methodology [4]. The approach is useful to motivate and engage adolescents, which is an essential requirement for systems’ acceptance and efficacy, rather than forcing people to accommodate technologies, products, or services. It should also be underlined that PEGASO, as a solution for prevention, is addressed to all people, independently of the presence or lack of the pathology. As a matter of fact, prevention specifically means tackling risk factors that are recognized in the scientific community as probability multipliers in directly or indirectly causing the specific pathology. This is a very difficult challenge because it is addressed to people who do not have a specific disease or even a minimal sign of it. Indeed, preventative actions are aiming at, e.g., introducing healthy habits in nutrition, adopting an active lifestyle, and minimizing the use of smoking, alcohol or other substances, i.e., reducing all the possible factors that in the short or long term can contribute to the development of cardiovascular or metabolic diseases. This action is recognized as the key strategy for future health care, not only to minimize the diffusion of these illnesses but above all to assure a good, long and active life for people and to reduce the costs of the health care systems, contributing to the overall economic sustainability of the welfare systems.

Preventative actions can be undertaken at different levels: (a) education; (b) monitoring; and (c) awareness. As a voluntary action, prevention is to be induced by an engagement strategy: in this sense, UCD is a winning method. By addressing user needs and preferences, it is easier for products and systems to be adopted by the same people contributing to their design. This is an application pull approach that represents the other side of the innovation strategy. Usually in sensor development, the technology-driven approach (also called the technology push method) is followed: it means starting from the technology and its requirements to design the final system. Instead, UCD starts from the users’ needs and preferences to define the functional and technological specifications that the system has to meet. The main advantage of this methodology is the high user acceptance and the usability of the final product. In these types of applications, such as prevention, this issue is fundamental for the long-term adoption of these systems.

Lifestyle monitoring solutions [5] and their integration into wearable accessories and smart clothing has emerged as ICT solutions, able to capitalize on the latest advances in sensing, signal analysis and communications [6,7,8,9,10,11,12]. This has generated a number of commercial products and experimental prototypes, demonstrating the important advances achieved in enabling ICT technologies. Nowadays, wearable sensors and smart textiles have become key elements of the lifestyle coaching service in an ecological monitoring setting for a single user–responsive system. Nevertheless, the available commercial products often offer limited functionality and accuracy in representing reliable monitoring solutions, while experimental prototypes are obstructive, conspicuous, require skilled users, or only apply to specific populations. In both cases, a step forward has to be done to fulfil the mobility and ergonomic requirements crucial for their public acceptation and commercial exploitation.

Individual monitoring represents a key point in the PEGASO strategy. In the PEGASO frame, the main purpose of the sensing component of the platform is to collect data about teenagers’ physical activity and behavioural habits. The adoption of an active lifestyle and the monitoring of its related physical parameters in an engaging, social platform is one of the objectives of the technological platform of the project [3]. In this view, the design of a dedicated wearable platform represents a specific challenge and goal. As a matter of fact, some statistics affirm that there is a lack of continued utilization of these products, namely sports and activity monitors, limited to six months of use. To strengthen acceptance and continued use of the wearable devices by the users is a key aim of PEGASO. This objective could be reached by involving the users from the beginning of the device’s development, using a UCD approach.

**Figure 1:**
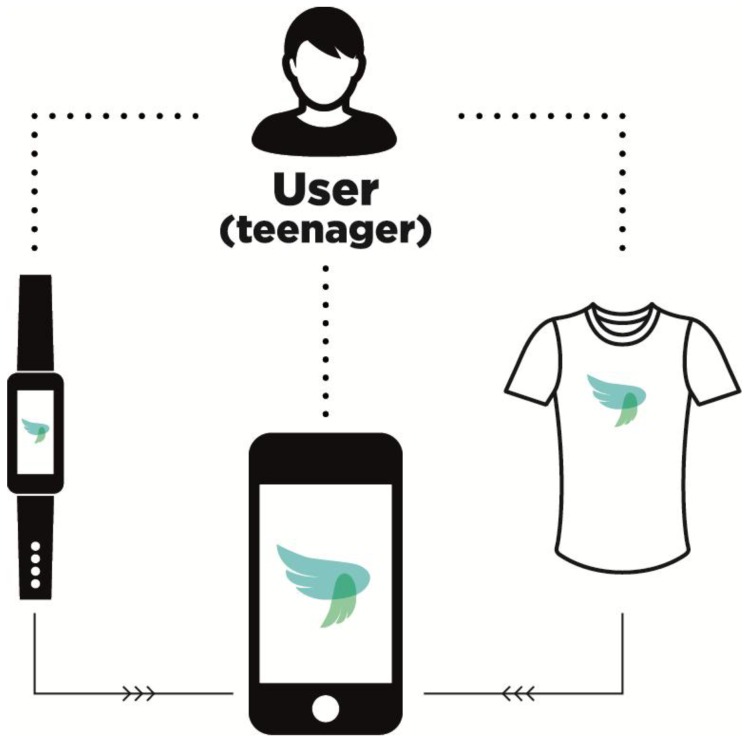
PEGASO sensors system.

The PEGASO system architecture is based on a mobile platform that collects and integrates data coming from physical parameters, healthy behaviour and environmental feedback. These data are collected using three levels of sensors (Figure 1). The first one is the smartphone, which represents the central and main important part of the system. Teens usually have and use smartphones. If the PEGASO apps are installed, the smartphone allows the functionality of the system and it is able to provide the minimum level of information that guarantees system operability. A smart wristband could be considered the second level of PEGASO sensors. This smart bracelet consists of an activity tracker that measures physical activities (thanks to a three-axis accelerometer) during the day and calculates energy expenditure. Users could wear it and all the data acquired are transferred to a dedicated app via Bluetooth. The wearable electronic system and the smart garments constitute the third level of the PEGASO sensor system. The wearable system is addressed to the short-term monitoring of physiological parameters during sport activities. The electronic module measures cardiac activity (with an I-lead ECG), physical activity (thanks to a three-axis accelerometer) and energy expenditure. The device is connected to the garments using a two-press stud and it sends the data acquired via Bluetooth. The smart garment is equipped with the textile electrodes at the chest level and it allows a continuous monitoring of the physiological parameters in an unobtrusive and comfortable way [6,7,8,9,10,11]. The textile electrodes are made of a 3D conductive textile, in which a silver yarn is mixed with a 3D static filament. This textile was chosen after several laboratory tests among different conductive textiles [13]. The tested textiles differ in the composite mixture of conductive and non-conductive yarns, which means a substantial change in terms of conductivity, elasticity and the properties’ decay after usage and washing cycles. Among different samples of conductive textiles, the 3D textile kept its properties after the laboratory test [13].

This paper presents and discusses the development of the wearable monitoring system and of the co-design activities conducted with teenagers, to define requirements and specifications at the functional, technical and aesthetic levels, and to increase its acceptance and compliance. Secondly, the paper focuses on the functional validation of the smart garments through the proposal of a first standardized protocol to verify the signal reliability with respect to some activities of daily living.

## 2. Materials and Methods

We recruited and cooperated with potential users in the design and development of devices and technologies, pointing out the features and functional advantages that the system offers. The use of a co-design activity represented an important step in the approach adopted in PEGASO because by making the final user actively participate in the design process, the final acceptance and compliance of the system could have more success and value. This was done by means of co-design focus groups in the first year of the project.

### 2.1. Co-Design Focus Groups

Since the beginning of PEGASO, teens were involved through focus groups in Italy, Spain, and the UK to elicit and understand their opinion about health, the importance of a healthy and active lifestyle, the use of mobile and social media for health purposes and the monitoring of their activities and physiological parameters through wearable devices. Teenagers were recruited through schools, focusing on fostering communities of interest (i.e., all students in a class), rather than students with identified risk factors.

Focus groups were methodologically organized into three phases. In total 407 European adolescents participated in the focus groups. Table 1 below shows the distribution of teenagers per country and per phase. Before recruitment, a dedicated informed consent was approved by each school institution and signed by the parents.

**Figure 2:**
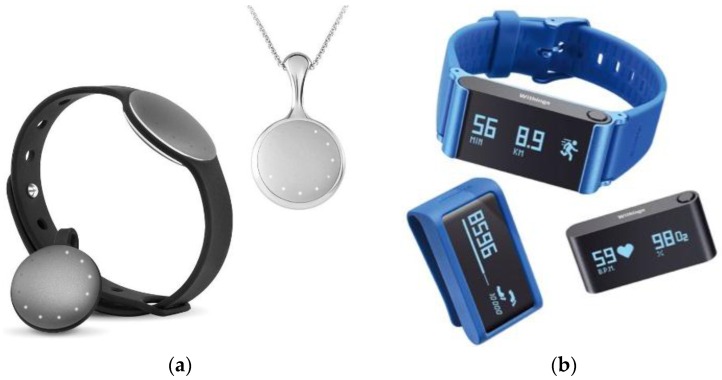
The commercial life trackers given to the users: (**a**) Misfit Shine; (**b**) Withings Pulse O_2_.

During the first phase or the focus group, the PEGASO Project was presented to teens, investigating their level of awareness about the use of technology for health purposes. The second phase was addressed to the exploration of sensors and wearable technologies for health management. Each participant tested one commercial life tracker for one week; different models were proposed, some stressing the design aspects and hiding the functionality (i.e., the Misfit Shine), some more evidently showing the functionality and the technology behind (i.e., the Withings Pulse). Figure 2 shows the devices used for the experiment. The goal was to collect participants’ opinions about the use of wearable sensors and their features.

In addition, some design samples of PEGASO garments (two sport t-shirts and two sport bras) were shown, to investigate the most important features that the teenagers consider relevant to improve the acceptance of the wearable apparel. The third phase was specifically dedicated to co-design activities as will be explained in detail in the next paragraphs.

**Figure 3:**
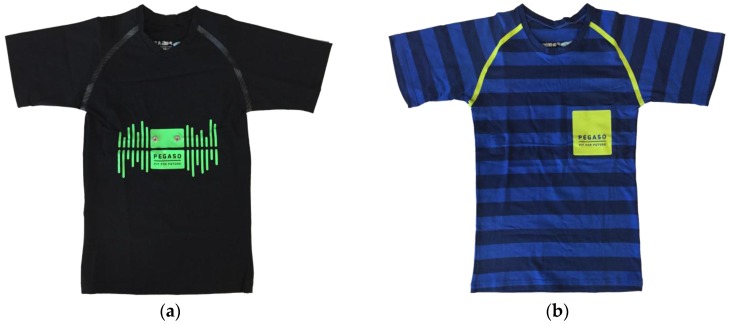
The comparison of the two male versions of the first set of sensorized t-shirts: (**a**) the style with technical textile and the visible device to be attached to the two snap buttons; (**b**) the style with a more fashionable colour and the sensing device hidden into the pocket where the two connecting snap buttons are placed.

**Figure 4:**
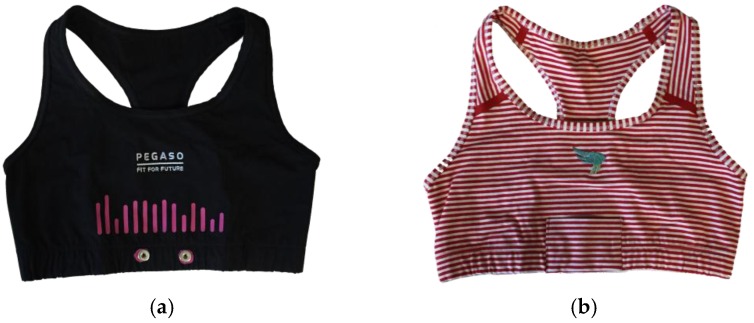
The comparison of the two female versions of the first set of sensorized bras: (**a**) the style with technical textile and the visible device to be attached to the two snap buttons; (**b**) the style with a more fashionable colour and the sensing device hidden into the pocket where the two connecting snap buttons are placed.

For the second phase, as a function of some preliminary preferences expressed by a sample of subjects interviewed at the beginning of the project in the first focus group phase, an initial set of sensing garments was proposed. The main issue underlined by teenagers is the willingness to show or to hide the sensing technology: some of them are technology “addicted” so they like to show to others that they are up to date with the most recent systems. Instead, a second group of adolescents indicated that they would like to wear a normal t-shirt equipped with sensing capabilities; this means that the sensors have to be embedded into the t-shirt and the sensing device has to be fixed and hidden by clothes items. According to these suggestions, two different samples of garments were developed and presented in the first focus group: one set of garment was made with the “visible sensors” concept, the other one following the “hidden sensors” vision. Figure 3 and Figure 4 compare the male and female versions of the two prototypes. 

The “Visible Sensor Line” was made of a technical textile, used for professional sport activities, in order to be comfortable and guarantee skin perspiration. High elasticity assures the proper sensor stability over the skin of the adolescent. The “Hidden Sensor Line” was made of jersey cotton, in order to look like everyday garments. The device was hidden by a pocket on the chest, laterally as in the male version or centrally as in the bra version. Two textile electrodes are embedded in both the smart garments: they allow the recording of the I-leading ECG signal, together with respiration; the sensing device also mounts a three-axis Inertial Monitoring Unit (IMU) to detect and measure activity and movements. The textile electrodes are made of silver-based yarn that demonstrated high electrical conductivity, stability of the sensors with respect to repeated washings and signal quality comparable to the one obtained through the standard silver/silver chloride adhesive medical electrodes. The smart garments are then connected with the electronic wearable device by means of two snap buttons.

**Figure 5:**
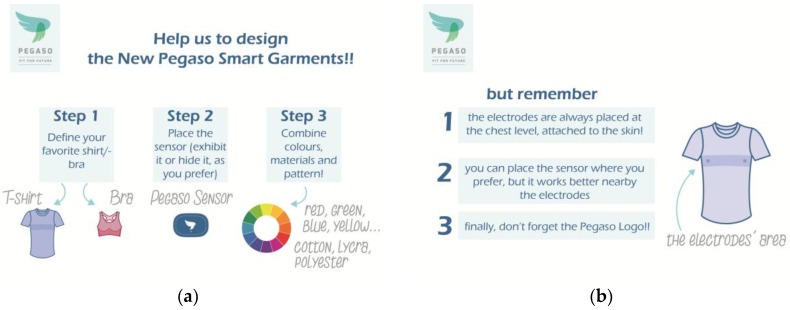
The co-design rules and related suggestions to comply with basic requirements: (**a**) the three-step development design activity explained to the teenagers; (**b**) the three fixed points imposed by the technical and functional requirements for signal monitoring and project dissemination.

In the third phase of the co-design activity, in a dedicated focus group, users were provided with a co-design template, and asked to sketch the new PEGASO smart garments. Some guidelines and suggestions were given, related to materials, colours or for driving the proper sensors’ positioning for the physiological measurements; they were then asked to design t-shirts, bras or vests (Figure 5).

### 2.2. Overall System Raw Signal Reliability Protocol

Following the aesthetic suggestions/requirements that emerged from the three focus group phases, the final garment prototypes were made. These prototypes were shown to the users, to get their comments and opinion about fabrics, design and aesthetic aspects. The smart garments were also tested in order to evaluate the signal reliability with respect to skin motion artefacts. Ten healthy volunteers (five males and five females) were recruited. They were asked to wear the smart garment (proper size and version—male/female—for each subject) and to connect the PEGASO wearable electronic device (WES). After that, they were asked to do some physical exercises and repeat three times each of the following experimental procedures. Table 2 below shows the phases of the testing protocol.

## 3. Results and Discussion

### 3.1. Focus Group Results

The focus groups gave the chance to explore the user attitude towards the introduction of wearable monitoring technologies into one’s life. Given the available technologies and the future perspectives, all the participants demonstrated a great interest in wearable sensors, considering them comfortable and useful for the monitoring of their lifestyle. Some of them said they would prefer to use these garments only during exercises, instead of during the entire day and in place of their clothes (“If I wanted something in my clothes I would rather wear a wristband that is more discrete”). They demonstrated interest in the garments’ price, their availability on the market, and information about the design, materials and colours. They were very curious about which data were detected using the garments, especially during their sport activities, so that they could monitor performance improvement. Another strong point was the comfort in the use of the garments with the embedded smart textile during sports activities compared with bracelets. Subjects, indeed, said they would be allowed to use these garments while playing sports (e.g., during the volleyball training sessions they cannot wear anything such as bracelets or necklaces, but they are allowed to wear smart garments).

Two models achieved resounding success among the 173 users interviewed: the most rated (42 of 173) was the black, waisted-shaped model; this t-shirt has a sporty design and tightens in the waist, with green seams. The second one (voted by 38 of 173 users) was a white t-shirt, with a smooth design, short and blue sleeves and a small purple pocket on the left side of the chest. 

**Figure 6:**
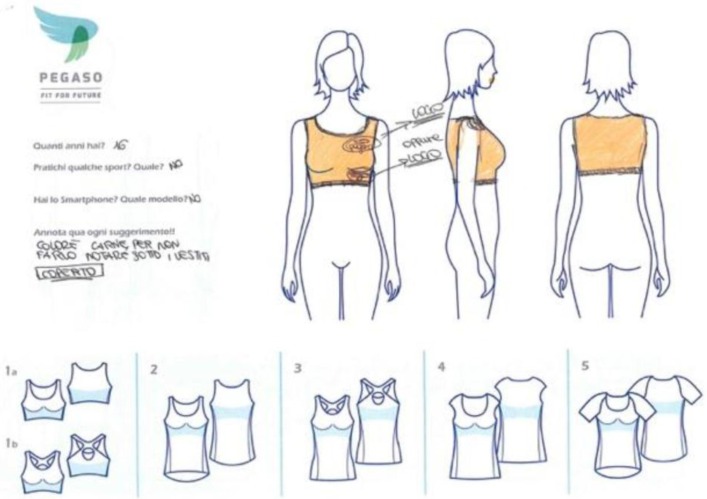
One example of the sketches produced by Italian teenagers for the co-design focus: the bra version according to the vision of a 15-year-old female student.

As result of the third focus group, teens gave feedback on the garments’ features and produced some sketches. They had to choose among several templates of t-shirts, bras and vests and design their favourite one. As in the previous phases, there was no unanimity on the sensor placement: some of them preferred the visible one, the others the hidden (Figure 6).

Concerning the co-design sketches, users outlined three different t-shirt models: males drew very colourful models, with symbols such as the peace sign or their favourite’s band logo; some of those were singlet models, and others were t-shirts with short sleeves. Females sketched solid colour, in particular black and white, bras to put under any t-shirt. All the interviewed users stated their preference for hidden sensors, embedded in the t-shirt and not visible to other people. Most of the subjects indicated that they would prefer light and technical fabrics, such as the ones already used for sport clothes.

Users were very interested in the monitoring features but they indicated the garments’ design as a point to take into high consideration, recommending us to collaborate with fashion designers. The aesthetics of the PEGASO system drive its acceptability.

Based on the results of the focus groups, the second generation of smart garments was developed and tested in the pre-pilot phase (i.e., a three-step validation period in preparation for the full pilot): in this phase, the potential users could experience a real interaction with PEGASO technologies. The pre-pilot is made of three steps, addressed to the evaluation of the devices and apps. The first step was related to testing and evaluating the PEGASO system in a controlled environment; mock-ups of the PEGASO apps were installed in users’ devices and tested and the game was shown in demo mode. Feedback was collected and this led to an adjusted design and graphics. The second step consisted of the real use of the PEGASO system. For one week, a group of users tested the devices (a wristband and a monitoring sensor), the garments and the apps. The last pre-pilot is still ongoing at the time of the writing of this paper.

As result of these two testing steps, the three main outcomes from the users’ feedback, in terms of usability and acceptability, are the following ones. (a) Users prefer to wear garments just during sports and training rather than during their usual daily activities. The participants liked the shirts and found them comfortable to wear, though they expressed the opinion that the design needs to be improved and made more fashionable; (b) They liked that the t-shirts were stretchy and that they fit all of them. Some of the participants expressed that they would have preferred other fabrics instead of those presented; (c) Short sleeves and tank top-style shirts were preferred. While wearing the sensors, participants did not feel restricted in their movements/activities at all and did not notice them.

### 3.2. System Reliability Results

Ten healthy volunteers tested the smart garment and the signal reliability with respect to skin motion artefacts while performing movements of the activities of daily living; the data are reported in Table 3. Users were asked to wear the smart garment, to connect the WES and to start performing a standardized set of defined exercises (as shown in Table 2 above). The data and signals acquired during the test were processed to compute the quality of the signal during each step according to the following criteria:

- Criterion 1: the ECG signal is suitable for processing to compute the main parameter (beat-to-beat heart rate and related secondary parameters, e.g., heart rate variability); scoring system: 0 = N, 1 = Y;
- Criterion 2: the percentage time of a high quality signal; scoring system: from 0 = 0% to 1 = 100%;
- Total score in each step for each subject = Criterion 1 * Criterion 2.

The test can be considered as successful if the overall score is equal or greater than 0.8 after the complete test.

The recruited subjects are described in the following table.

As shown by the results in Table 4, the most critical activity is demonstrated to be flexion/extension arm movements because they produce severe skin motion artefacts affecting the signal quality.

## 4. Conclusions

The results of this part of the study concern the methodology to develop innovative wearable sensors not simply from the technological point of view but, above all, in relation to users’ feedback and compliance. The case study was the PEGASO system technologies (garments, apps, game), which is one aspect of the entire project’s outcome.

Preliminary findings are related to the vision and judgments provided by adolescents about the new wearable monitoring technologies. The main interesting elements of the data collected during the focus groups and related analysis raised that the life shortness of activity trackers is a common topic in literature and the phenomenon is particularly widespread among teens. As a matter of fact, teens revealed they were interested in monitoring their activity and wearing smart clothes. The outcome is that designing devices answering their desiderata is really the challenge, and the aforementioned involvement strategy could be the key.

In fact, the UCD approach was demonstrated to be effective and reliable in providing solutions that match users’ requirements and preferences. This would increase the actual daily use of these wearable devices for implementing the prevention strategies adopted by the project in promoting healthy habits into the selected teenager cohort.

Moreover, an evaluation protocol for the smart garment and its signal reliability with respect to skin motion artefacts was defined. Thanks to the different exercises performed, the wearable system functionality can be tested in real usage. The system developed proves its reliability and the good quality of the signal acquired during usage. 

The PEGASO framework will be validated by secondary school students during the pilot test phase. Four validation studies will be carried out in Italy (Lombardy), Spain (Catalonia) and the United Kingdom (England/Scotland), involving about 400 students. The validation of the PEGASO platform will first assess the system and technology acceptance, and usability and long-term use. These factors will be assumed also as a secondary assessment of the motivation and engagement of teenagers in the adoption of prevention strategies. From a technology perspective, the pilot tests will also evaluate the reliability in assessing the teenagers’ lifestyles and their changes (with a focus on eating habits and on physical activities) and the related efficacy on the sensors’ network system. Also, at the end of the project we will be able to have a complete assessment of the efficiency of the system in encouraging lifestyle change.

## Figures and Tables

**Table 1:** Number of participants ^1^ in the focus groups in the three phases in each country.

Phase	Italy	Spain	United Kingdom
1	75	28	45
2	27	30	14
3	66	28	94
Total per Country	168	86	153
Total per Project		407	

^1^ Adolescents (male and female) aged 13–17.

**Table 2:** Overall system raw signal reliability protocol.

Phase	Description	Figure
0	Rest for 20 s with arms at the sides	
1	20 times: flexion/extension arms movements (10 left + 10 right)	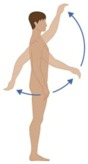
2	20 times: abduction/adduction arms movements (10 left + 10 right)	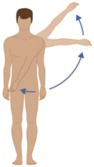
3	20 times: inside/outside arms movements on the horizontal plane (10 left + 10 right)	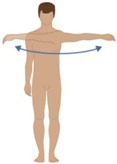
4	10 times: elevation and depression of the shoulders	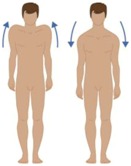
5	10 times: retro/anteposition of the shoulders from back to front	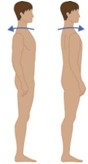
6	10 times: rotation of the torso from left to right	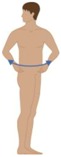
7	10 times: flexion/extension of the torso from back to front (10 front + 10 back)	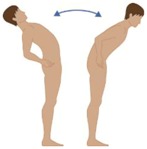
8	10 times: right and left lateral flexion of the torso from left to right (10 left + 10 right)	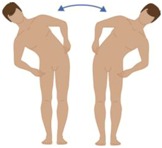

**Table 3:** Participants’ characteristics.

Subject ID	H (cm)	W (kg)	Age (Years)	Sex
1	186.0	105.2	46	M
2	155.5	56.2	33	F
3	166.5	52.9	33	F
4	157.0	53.0	34	F
5	168.5	68.9	33	F
6	173.0	68.8	53	M
7	178.0	85.6	33	M
8	171.0	64.5	29	F
9	181.5	79.6	51	M
10	174.5	68.7	63	M

**Table 4:** Signal reliability scores.

Phase Subject	0	1	2	3	4	5	6	7	8	Total
s1	1	0.7	0.6	0.7	1	0.8	1	1	1	0.87
s2	1	0.8	1	1	1	1	1	1	1	0.98
s3	1	0.2	0.8	0.2	0.7	1	1	0.3	1	0.69
s4	1	0.8	1	1	1	1	1	1	1	0.98
s5	1	0.7	0.8	0.8	0.8	0.8	0.8	0.8	1	0.83
s6	1	0.7	1	0.8	1	1	1	0.8	1	0.92
s7	1	1	1	1	1	1	1	1	1	1
s8	1	1	1	1	1	1	1	1	1	1
s9	1	0.9	1	1	1	1	1	0.5	1	0.93
s10	1	1	1	1	1	1	1	1	1	1
avg	1	0.78	0.92	0.85	0.95	0.96	0.98	0.84	1	0.92
sd	0	0.24	0.14	0.25	0.11	0.08	0.06	0.25	0	0.1

## Abbreviations

The following abbreviations are used in this manuscript:

UCD	User-Centred Design
ICT	Information and Communication Technologies
ECG	Electrocardiogram
ICT	Information and Communication Technologies
IMU	Inertial Monitoring Unit
EU	European Union
FP7	7th Framework Programme
WES	Wearable Electronic System
